# The complete mitochondrial DNA analyses of Inner Mongolia Cashmere goat type of Erlangshan species

**DOI:** 10.1080/23802359.2017.1419084

**Published:** 2017-12-22

**Authors:** Yuhao Ma, Rui Su, Yixing Fan, Xian Qiao, Xiaokai Li, Lei Zhang, Jinquan Li

**Affiliations:** a College of Animal Science, Inner Mongolia Agricultural University, Hohhot, China;; b Key Laboratory of Animal Genetics, Breeding and Reproduction, Inner Mongolia Autonomous Region, Hohhot, China;; c Key Laboratory of Mutton Sheep Genetics and Breeding, Ministry of Agriculture, Hohhot, China;; d Engineering Research Center for Goat Genetics and Breeding, Inner Mongolia Autonomous Region, Hohhot, China

**Keywords:** Inner Mongolia Cashmere goat, Erlangshan Cashmere goat, mitogenome, phylogenetic

## Abstract

Inner Mongolia Cashmere goat is evolved from Mongolia goat in long-term breeding, as a source of high-quality cashmere, Inner Mongolia Cashmere goat type of Erlangshan is famous in the world. By using molecular biology techniques, we sequenced the complete mitochondrial DNA of Erlangshan Cashmere goat. The complete length of sequence is 16,640 bp, containing 13 protein-coding genes, 22 transfer RNA genes, two ribosomal RNA genes and a control region (D-loop region). Our mitogenome will enlarge genomic information for further studies on evolution and domestication of Inner Mongolia Cashmere goat, enhance germplasm conservation and breeding programs of domestic goat.

Goat is one of the most important and widespread domestic animal, has a great ability to adapt environment and provides many economic productions such as cashmere, leather and meat. Researchers analyzed evolution of goat and found it was developed from the bezoar (Capra *aegagrus*) in the Fertile Crescent (Naderi et al. 2008). China is the largest cashmere production country in the world and about 30% products are from Inner Mongolia Autonomous Region. Inner Mongolia Cashmere goat is evolved from Mongolia goat in long-term breeding, known for its fantastic cashmere production trait including longer fibre and short diameter (Zhou et al. 2003), and was included in the first released protection list of animal genetic resources. As a result of selective breeding and improvement, Inner Mongolia Cashmere goat type of Erlangshan is characterized by a great performance of cashmere (Wudubala et al. 2016).

In this study, we reported the whole mitogenome of Erlangshan Cashmere goat and the sequence was deposited into GenBank under the accession number MF573068. We extracted whole genome sample from a Erlangshan Cashmere goat’s blood which collected from Bayan Nur City (east longitude 105°C12′–109°C59′ north latitude 40°C13′–40°C28′). First, the genome sequence library was constructed, connected the A&B connector and produced single-stranded DNA fragments. Bridge PCR was used to amplify mitochondrial DNA and was to obtain the sequence of the template DNA fragment sequenced by Illumina HiSeq 2000 by Genesky Biotechnologies Inc. Shanghai 201315, China. The sequence was assembled by mitoMaker software and analyzed the phylogenetic with Inner Mongolia Cashmere goat type of Alashan and Aerbasi, Liaoning Cashmere goat, San Clemente goat. The cover degree of the sequenced genome and gene regions was more than 95% and 98%, respectively. The mistake rate of single basic group was under the hundred thousandth. The mitogenome is 16,640 bp in length, contains 37 genes, two ribosomal RNA genes (12S and 16S rRNA), 13 protein-coding genes (PCGs), 22 transfer RNA genes and a D-Loop region. The overall A + T content of the whole mitogenome is 60.84%.

According to phylogenetic tree analysis, Erlangshan goat has a closer genetic relationship with Alashan Cashmere goat, and further genetic distance with San Clemente goat (Figure 1).

**Figure 1.**
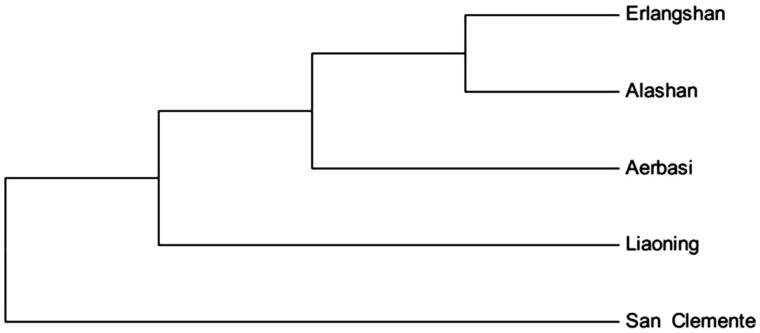
Phylogenetic tree of Cashmere goat, based on the complete mitochondrial DNA sequences analysis. Inner Mongolia Cashmere goat includes Erlangshan, Alashan and Aerbasi types. Liaoning is Liaoning Cashmere goat, San Clemente is San Clemente goat.

In this study, we provided the mitogenome sequence of the Erlangshan Cashmere goat, it will be a significant for further taxonomic classification, phylogenetic reconstruction and conservation strategies of the endemic species.
